# Genome engineering and direct cloning of antibiotic gene clusters via phage ϕBT1 integrase-mediated site-specific recombination in *Streptomyces*

**DOI:** 10.1038/srep08740

**Published:** 2015-03-04

**Authors:** Deyao Du, Lu Wang, Yuqing Tian, Hao Liu, Huarong Tan, Guoqing Niu

**Affiliations:** 1 State Key Laboratory of Microbial Resources, Institute of Microbiology, Chinese Academy of Sciences, Beijing, China; 2 University of Chinese Academy of Sciences, Beijing, China; 3 Key Laboratory of Industrial Fermentation Microbiology, Ministry of Education, College of Biotechnology, Tianjin University of Science and Technology, Tianjin, China

## Abstract

Several strategies have been used to clone large DNA fragments directly from bacterial genome. Most of these approaches are based on different site-specific recombination systems consisting of a specialized recombinase and its target sites. In this study, a novel strategy based on phage ϕBT1 integrase-mediated site-specific recombination was developed, and used for simultaneous *Streptomyces* genome engineering and cloning of antibiotic gene clusters. This method has been proved successful for the cloning of actinorhodin gene cluster from *Streptomyces coelicolor* M145, napsamycin gene cluster and daptomycin gene cluster from *Streptomyces roseosporus* NRRL 15998 at a frequency higher than 80%. Furthermore, the system could be used to increase the titer of antibiotics as we demonstrated with actinorhodin and daptomycin, and it will be broadly applicable in many *Streptomyces*.

S*treptomyces* are high-GC Gram-positive bacteria well known for their ability to produce a wide variety of medically and agriculturally useful antibiotics and related compounds^1^. Genes responsible for the biosynthesis of a specific secondary metabolite are usually arranged in clusters that vary in size from a few to over 100 kb^2^. To gain insight into the biosynthesis and regulation of antibiotics in *Streptomyces*, it is of great importance to clone their gene clusters. Recently, various approaches have been developed to clone gene clusters directly from bacterial genomic DNA. These methods include RecET-mediated linear-plus-linear homologous recombination (LLHR)^3^, *oriT*-directed capture system^4^ and transformation-associated recombination (TAR)^5^. The RecET-mediated LLHR was successful in cloning gene clusters (10 to 52 kb in length) from the genome of *Photorhabdus luminescens* into expression vectors in *Escherichia coli*^3^. The *oriT*-directed capture system has been used to clone regions up to 140 kb from the genome of *Burkholderia pseudomallei*^6^ and 200 kb from megaplasmid of *Sinorhizobium meliloti*^4^. However, the use of this system was limited to Gram-negative bacteria that can be established as conjugation donors^6^. Taking advantage of the natural *in vivo* homologous recombination of *Saccharomyces cerevisiae*, TAR cloning strategy was used to capture a 21.3 kb enterocin gene cluster from *Salinispora pacifica* CNT-150^7^ and a 67 kb taromycin A biosynthetic gene cluster from *Saccharomonospora* sp. CNQ-490^8^.

The ability to delete large genomic fragments within *Streptomyces* genome is of great interest for genetic manipulations of *Streptomyces*. Several strategies have been developed for a number of bacteria. Some methods are based on the meganuclease I-SceI system which involves the meganuclease I-SceI of *Saccharomyces cerevisiae* and its 18 bp recognition sequence^9^,^10^. Many of them are based on site-specific recombination systems consisting of a specialized recombinase and its target sites. Nearly all site-specific recombinases fall into two families, the tyrosine recombinases and the serine recombinases^11^. The recombination systems of the tyrosine recombinase family include Cre/loxP from the P1 phage^12^, Dre/rox from the P1-like transducing phage D6^13^ and the Flp/FRT from yeast^14^. The Cre, Dre and Flp proteins are the tyrosine recombinases which catalyze reciprocal site-specific recombination of DNA at loxP, rox and FRT sites, respectively. Integrases (Int) from *Streptomyces* temperate phage ϕC31 and ϕBT1 belong to serine recombinase family. They catalyze site-specific recombination of the phage attachment site (*attP*) with the bacterial attachment site (*attB*), resulting in the formation of two hybrid sites (*attL* and *attR*)^15^,^16^. Both ϕC31 and ϕBT1 *attP*-*int* loci have been used to construct versatile vectors which can integrate into different *attB* sites in *Streptomyces*^15^,^16^. To increase the diversity of *attP*-*attB* pair of ϕBT1, 15 mutated *attP*-*attB* pairs (*attP_01_*-*attB_01_* → *attP_15_*-*attB_15_*) were generated by PCR mutagenesis of the central dinucleotide sequence of *attB* and *attP*^17^. The Cre/loxP system was successfully used for the deletion of large fragments in *Magnetospirillum gryphiswaldense* and several *Streptomyces* species^18^,^19^,^20^. However, the use of ϕC31 and ϕBT1 integrase in this aspect has not been exploited.

We devised a novel strategy for *Streptomyces* genome engineering and cloning of antibiotic gene clusters. This method is based on phage ϕBT1 *attP*-*attB*-*int* system and requires two single crossovers for targeted integration of mutated *attB* and *attP* into the recipient chromosome. Using the system, we easily cloned 25 kb fragment containing actinorhodin (*act*) gene cluster from *S. coelicolor* M145, 45 kb fragment containing napsamycin (*nap*) gene cluster and 157 kb fragment containing daptomycin (*dap*) gene cluster from *S. roseosporus* NRRL 15998. In addition, this method could be used to improve the titer of antibiotics by increasing copy numbers of antibiotic gene clusters.

## Results

### Construction of pUC119- and pKC1139-based plasmids

**Figure 1:**
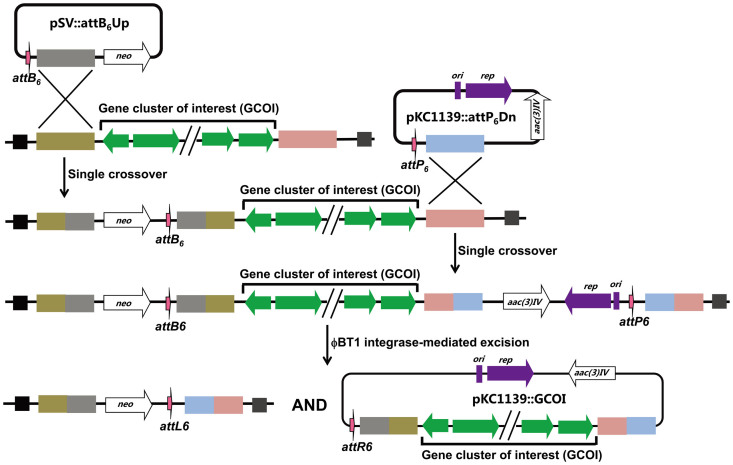
Schematic diagram of antibiotic gene cluster cloning from *Streptomyces* chromosome. Initially, a pUC119-based suicide plasmid (pSV::attB_6_Up) carrying *attB_6_* and a region homologous to 5′ end of the cluster is introduced into the chromosome by a single crossover. A second plasmid pKC1139::attP_6_Dn is based on pKC1139 carrying *attP_6_* and a region homologous to 3′ end of the cluster. When the incubation temperature is higher than 34°C, pKC1139::attP_6_Dn turns into a non-replicating plasmid and then is integrated into the chromosome by a single crossover. Expression of ϕBT1 integrase (encoded in the plasmid pIJ10500) leads to excision of the pKC1139 backbone with gene cluster of interest, leaving behind the suicide vector pUC119::*neo* and 42 bp *attL6* site. *aac(3)IV*: apramycin resistance gene; *neo*: kanamycin resistance gene; *ori*: temperature-sensitive origin of replication from pSG5; *rep*: *rep* encoding a replication initiator protein from pSG5.

Our strategy used in this study requires both homologous and site-specific recombinations. The homologous recombinations were used for targeted integration of the mutated *attB* and *attP* into *Streptomyces* chromosome, while the ϕBT1 integrase-mediated site-specific recombination was employed to excise targeted region of interest from the chromosome (Fig. 1). The mutated *attB* and *attP* sites were chosen to avoid site specific recombination with the endogenous *attB* site in *Streptomyces* genome and consequently undesirable DNA rearrangements. Sites of *attB_6_* and *attP_6_* were randomly chosen from the 15 mutated *attP*-*attB* pairs. For the integration of *attB_6_* into *Streptomyces* chromosome, pUC119-based suicide plasmids (pSV::*attB_6_*-*act*, pSV::*attB_6_*-*nap* and pSV::*attB_6_*-*dap*) were constructed. These plasmids are derivatives of pUC119 containing the kanamycin-resistance gene (*neo*), the origin of transfer (*oriT*) from plasmid RK2 (for the intergeneric conjugation between *E. coli* and *Streptomyces*), *attB_6_* and a 2.0 kb homologous region flanking 5′ end of the targeted regions (Fig. S1a). We also constructed pKC1139-based plasmids (pKC1139::*attP_6_*-*act*, pKC1139::*attP_6_*-*nap* and pKC1139::*attP_6_*-*dap*) for the integration of *attP_6_* into *Streptomyces* chromosome. These plasmids are derivatives of pKC1139 containing *attP_6_* and a 2.0 kb homologous region flanking 3′ end of the targeted regions (Fig. S1b).

### Cloning of *act* gene cluster from *S. coelicolor* M145

**Figure 2:**
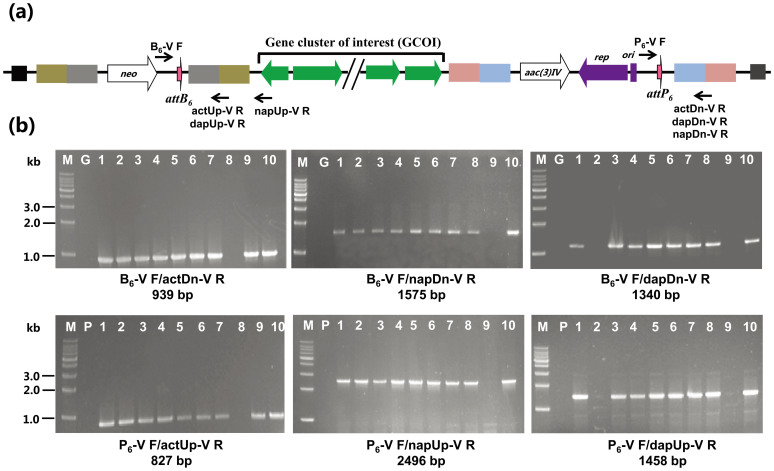
Confirmation of the excision events by PCR amplifications. (A) The schematic diagram showing the position of primers in the chromosome of double-cointegrate strains. (B) Agarose gel electrophoresis showing PCR amplified fragments. PCR templates in the upper panels are genomic DNAs from *S. coelicolor* M145 or *S. roseosporus* NRRL 15998 (G) and ten randomly selected double-cointegrate strains with pIJ10500 (M145-MCact, Sro-MCnap or Sro-MCdap), while PCR templates in the lower panels are plasmid DNAs including pKC1139 (P) and ten different clones of pKC1139::*act*, pKC1139::*nap* and pKC1139::*dap*. The primers used and the expected size of amplification fragments were indicated.

**Figure 3:**
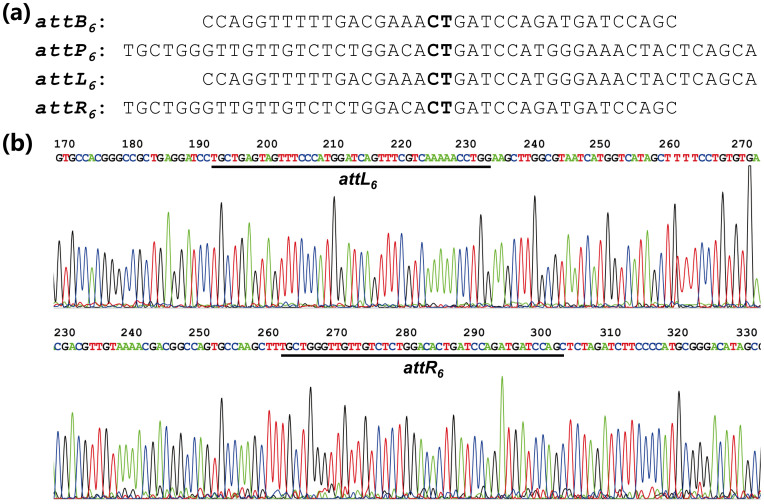
Representative excision of the *act* gene cluster from *S. coelicolor* M145. (A) Nucleotide sequence of *attB_6_*, *attP_6_*, *attL_6_* and *attR_6_*. The mutated core dinucleotide (CT) at which the crossover occurs is in bold. (B) Verification of *attL_6_* and *attR_6_* by DNA sequencing. Sequences of *attL_6_* and *attR_6_* from DNA sequencing are underlined.

To test this strategy, we first chose to clone the well-studied *act* gene cluster (*SCO5070*-*SCO5092*) from *S. coelicolor* M145. For this purpose, pSV::*attB_6_*-*act* and pKC1139::*attP_6_*-*act* were introduced into the recipient chromosomes via single-crossover homologous recombination to obtain double-cointegrate strain Sco-actB_6_P_6_ (Fig. 1). Further introduction of pIJ10500 (an integrative plasmid containing the ϕBT1 integrase gene) into Sco-actB_6_P_6_ allowed subsequent excision of 23 kb *act* gene cluster from *S. coelicolor* M145, leaving behind the suicide vector pUC119::*neo*, a scar of 42 bp *attL* site and pIJ10500 integrated within SCO4848. Excision of the gene cluster was confirmed by PCR analysis using both genomic and plasmid DNA as templates (Fig. 2). For 9 out of 10 exconjugates tested, the ϕBT1 integrase-mediated excision of *act* gene cluster occurred at a frequency of 90%. Furthermore, the presence of *attL* and *attR* in the amplified fragments was confirmed by DNA sequencing (Fig. 3). To recover the plasmid containing the entire *act* gene cluster (pKC1139::*act*) from *Streptomyces*, the DNA extract containing pKC1139::*act* from M145-MCact was used to transform *E.coli* Top10. Plasmid DNA from four apramycin resistant *E. coli* colonies was confirmed by BamHI and NotI digestion, respectively. The restriction fragments showed correct band patterns (Fig. S2a).

**Figure 4:**
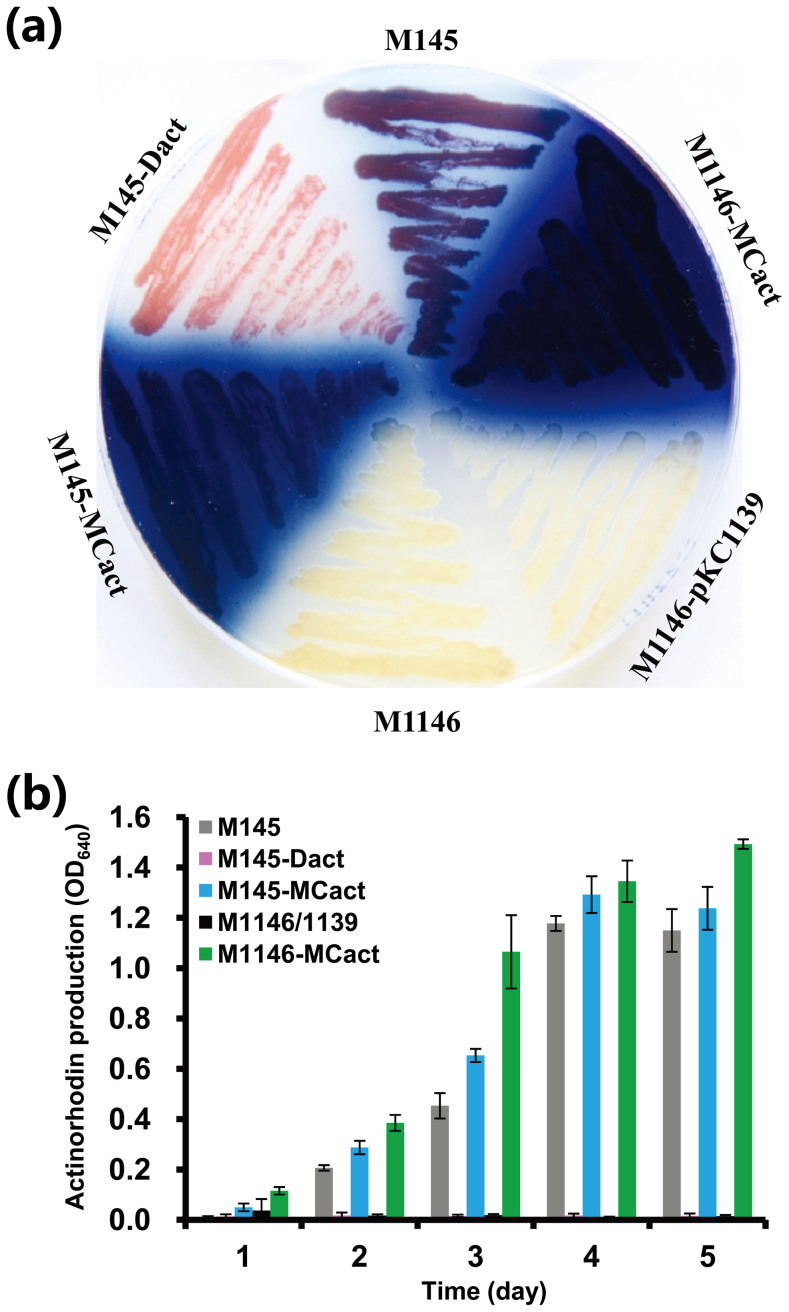
Comparison of actinorhodin production in *S. coelicolor* M145 and its derivatives. (A) Comparison of actinorhodin production (blue pigment) of *S. coelicolor* M145 and its derivatives. Photograph was taken from the bottom of the plate after grown on R5MS agar medium for 4 days at 28°C. Representative image of three independent experiments with similar results was shown. (B) Actinorhodin titers of *S. coelicolor* M145 and its derivatives grown in 50 ml of R5MS at 28°C. Error bars show standard deviations.

To verify the cluster is complete, the recombinant plasmid pKC1139::*act* was introduced into *S. coelicolor* M1146 (M1146) to obtain M1146-MCact. Unlike M1146 and M1146-pKC1139 (*S. coelicolor* M1146 containing empty vector pKC1139), M1146-MCact regained the ability to produce the blue pigment actinorhodin (Fig. 4a). These results showed that the cloned *act* gene cluster was complete and functional.

### Deletion of *act* gene cluster from *S. coelicolor* M145

To delete the *act* gene cluster from *S. coelicolor* M145, a single colony of M145-MCact was randomly chosen for the removal of pKC1139::*act*. After three rounds of nonselective growth at 28°C and subsequent cultivation at 40°C, approximately 5% of M145-MCact colonies lost pKC1139::*act*. Strains lacking the *act* gene cluster (M145-Dact) were first confirmed by PCR (data not shown), and then patched on R5MS solid agar plate for visual comparison of actinorhodin production. Unlike *S. coelicolor* M145 that could produce both blue pigment actinorhodin and red pigment undecylprodigiosins, M145-Dact could only produce the red pigment undecylprodigiosins (Fig. 4a). This was further validated by no actinorhodin production of M145-Dact in R5MS liquid culture (Fig. 4b).

### Cloning and deletion of *nap* and *dap* gene cluster from *S. roseosporus* NRRL 15998

To clone gene cluster of medium and large sizes, we used the same strategy to clone *nap* and *dap* gene cluster from *S. roseosporus* NRRL 15998. Excision of *nap* gene cluster from *S. roseosporus* NRRL 15998 occurred in 9 out of 10 exconjugates, and excision of *dap* gene cluster from *S. roseosporus* NRRL 15998 occurred in 8 out of 10 exconjugates (Fig. 2). Like pKC1139::*act*, plasmid containing *nap* gene cluster (pKC1139::*nap*) was passed through *E.coli* Top10 and isolated plasmid DNA was confirmed by BglII and EcoRI digestion, respectively (Fig. S2b). The cloned fragment covers a contiguous DNA region of 45 kb from *SSGG02973* to *SSGG03009*. For pKC1139::*dap*, the plasmid was isolated directly from *Streptomyces* and confirmed with restriction digestion (Fig. S2c). The 157 kb fragment covering *SSGG00215*-*SSGG00287* contains the complete *dap* gene cluster. Similar to that of *act* gene cluster, the removal of pKC1139::*nap* and pKC1139::*dap* from Sro-MCnap and Sro-MCdap generated strains lacking *nap* and *dap* gene clusters (Sro-Dnap and Sro-Ddap).

### Improvement of antibiotic titers

**Figure 5:**
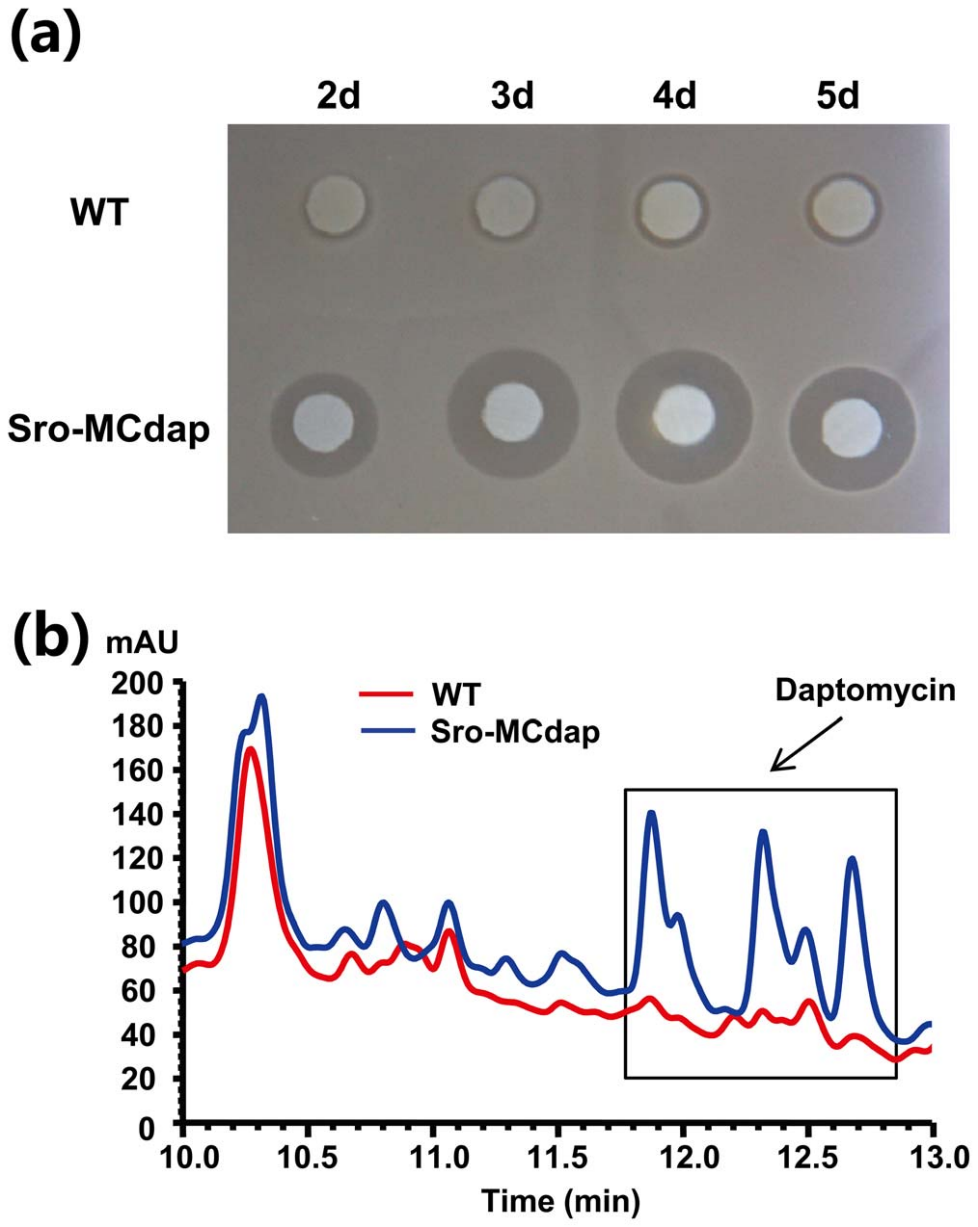
Analysis of daptomycin production in *S. roseosporus* NRRL 15998 (WT) and Sro-MCdap. (A) Bioassay of daptomycin against *S. aureus*. After grown on AS-1 agar for 2–5 days at 28°C, the patches of WT and Sro-MCdap were overlaid with cultures of *S. aureus* and the zone of inhibition was assessed after overnight incubation at 37°C. Representative images of three independent experiments with similar results are shown. (B) HPLC analysis of fermentation filtrates from WT and Sro-MCdap after incubation for 4 days. Components of daptomycin were indicated by comparison with standards.

The pKC1139 contains a temperature-sensitive origin of replication from pSG5, which is a medium copy plasmid with an approximate 20–50 copy numbers per chromosome^21^. When cultured at 28°C, pKC1139 exists as autonomous plasmid in *Streptomyces*. In *S. coelicolor* M145, there is only one copy of *act* gene cluster in the chromosome. After the ϕBT1 integrase-mediated excision, the *act* gene cluster was transferred into pKC1139. An increase in copy number of *act* gene cluster will improve actinorhodin production. This was confirmed both on R5MS agar plate (Fig. 4a) and in R5MS liquid culture (Fig. 4b). It should be noted that the titer of actinorhodin in M1146-MCact was even higher than that of M145-MCact. Similarly, daptomycin titer could also increase after the excision of *dap* gene cluster from its chromosome location in *S. roseosporus* NRRL 15998. Cultures of Sro-MCdap and *S. roseosporus* NRRL 15998 were subjected to bioassay against *S. aureus*, the results showed that Sro-MCdap exhibited bigger inhibition zones against *S. aureus* than *S. roseosporus* NRRL 15998 at time intervals from 2–5 days (Fig. 5a). This was further verified by comparison of daptomycin from fermentation broth of *S. roseosporus* NRRL 15998 and Sro-MCdap by high-performance liquid chromatography (HPLC) analysis (Fig. 5b). In addition, we noticed that existence of extra copy numbers of antibiotic gene clusters caused a slowdown in growth of *Streptomyces*. When cultured on AS-1 agar medium, growth of Sro-MCnap and Sro-MCdap are severely impaired, especially at earlier stages of cultivation (Fig. S3). This phenotype was most likely attributed to the metabolic burden of extra copy numbers of antibiotic gene clusters. This assumption is based on the observation that growth of Sro-Dnap (devoid of *nap* gene cluster) and Sro-Ddap (devoid of *dap* gene cluster) are converted back to that of *S. roseosporus* NRRL 15998 (Fig. S3).

To examine the stability of multiple copy plasmids in *Streptomyces*, two randomly chosen strains of Sro-MCdap were passed consecutively for five or ten times on AS-1 plates supplemented with or without apramycin. Biological activities of these stains (G_5_ and G_10_) were compared with that of the original Sro-MCdap (G_0_). All Sro-MCdap strains exhibited similar inhibitory activity against *S. aureus* (Fig. S4), suggesting that pKC1139-derived large plasmids are stable in the engineered *Streptomyces* in the presence or absence of selective pressure.

## Discussion

We have established an efficient method for genome engineering and direct cloning of gene clusters in *Streptomyces*. The strategy is based on phage ϕBT1 *attP*-*attB*-*int* system and provides several advantages over similar methods. First, it can be used for the deletion of large fragment (up to 157 kb) from *Streptomyces* genome. In the meantime, the large fragment containing gene cluster of interest was cloned into pKC1139 simultaneously. Another advantage of our strategy is that it could clone gene cluster in size up to 157 kb. This is the largest size ever reported in Gram positive bacteria and should be good enough for most antibiotic gene clusters. Last, our strategy can be used to improve the titer of industrial important antibiotics by creating strains with extra copy numbers of antibiotic biosynthetic gene clusters.

The ϕBT1 *attP*-*attB*-*int* system is helpful for genetic modifications of *Streptomyces* genome at multiple sites. In addition to the intact *attP*-*attB* pair, there are 15 mutated *attP*-*attB* pairs which can be recognized by ϕBT1 integrase^17^. Multiple rounds of large fragment deletion can be achieved with the following modifications. (1) Relocation of the *attB*_6_ sequence (or any other mutated *attB*) to the downstream of the 2.0 kb homologous fragment in pSV::attB_6_Up. This change will allow the excision of the pUC119::*neo* backbone from *Streptomyces* genome together with pKC1139. (2) Construction of an autonomous helper plasmid containing a temperature-sensitive origin of replication from pSG5, the origin of transfer (*oriT*) from plasmid RK2 and ϕBT1 integrase gene. This plasmid can ensure the high efficient excision of large fragment from *Streptomyces* genome and subsequent removal of ϕBT1 integrase. With these modifications, there is only 42 bp *attL* site left in the chromosome of *Streptomyces*.

Genome analysis suggested that *S. roseosporus* NRRL 15998 has potential capacity to produce napsamycins^22^,^23^. However, the production of napsamycins in *S. roseosporus* NRRL 15998 has not been reported. With this strategy, we cloned *nap* gene cluster in pKC1139 to generate pKC1139::*nap*. It can be manipulated extensively in *E. coli*. These manipulations include replacement of vector backbone with integrative plasmid and deletion or constitutive expression of regulatory gene by PCR targeting^24^. The modified gene cluster can be transferred into heterologous hosts for expression. It can also be transferred back into the mutant devoid of *nap* gene cluster after removal of pKC1139::*nap*. Detection of napsamycin in these strains will shed light on the activation of cryptic gene clusters in *Streptomyces*.

In some industrial overproducing strains generated by traditional mutagenesis, amplification of biosynthetic gene cluster has been observed^25^,^26^,^27^. Based on these observations, controlled amplification of gene cluster was used to increase the productivity of commercially important antibiotics. Integration of an additional copy of gene cluster for nikkomycin and gougerotin biosynthesis led to an increased production of nikkomycin and gougerotin by *Streptomyces ansochromogene*^28^ and *Streptomyces graminearus*^29^, respectively. The *zouA*-mediated gene amplification of *act* gene cluster in *S. coelicolor* M145 led to a 20-fold increase in actinorhodin production^30^. The *zouA* encodes a site-specific relaxase similar to TraA protein which catalyzes RecA-independent site-specific recombination. The recombination sites of ZouA are *oriT*-like RsA and RsB^30^. In this study, we reported the amplification of gene clusters mediated by phage ϕBT1 integrase and improved antibiotic titers in the engineered *Streptomyces* strains. We believe that the system described here could be used readily to increase antibiotic titers in many *Streptomyces* and possible other actinomycetes.

## Methods

### Bacterial strains, plasmids, primers and growth conditions

Bacterial strains and plasmids used in this study are listed in Table 1, and primers are listed in Table S1. *S. coelicolor* M145 and *S. roseosporus* NRRL 15998 were used for cloning of *act*, *nap* and *dap* gene clusters. *S. coelicolor* M1146 is an engineered derivative of *S. coelicolor* M145 that lacks gene clusters for actinorhodin (ACT), undecylprodigiosins (RED), cryptic polyketide (CPK) and calcium-dependent antibiotic (CDA) biosynthesis^31^. *Staphylococcus aureus* was used as an indicator strain for daptomycin bioassay. *E. coli* Top10 was used as a general host for propagating plasmids. *E. coli* ET12567 (pUZ8002) was used as a host for transferring DNA from *E. coli* to *Streptomyces* by intergeneric conjugation^32^.

For general purpose, *S. coelicolor* M145 and its derivatives were grown on mannitol soya flour medium (MS) agar or in yeast extract-malt extract (YEME) liquid medium^32^. For actinorhodin production, *S.* *coelicolor* M145 and its derivatives were grown on R5MS agar or in R5MS liquid medium^33^. *S. roseosporus* NRRL 15998 was cultured on AS-1 agar medium or in tryptic soy broth (TSB) liquid medium^34^. All *Streptomyces* stains were maintained at 28°C unless specified otherwise. General approaches for *E. coli* or *Streptomyces* manipulations were performed according to standard protocols^32^,^35^. When necessary, the final antibiotic concentrations used for selection of *E. coli* transformants were as follows: ampicillin, 100 μg ml^−1^; apramycin, 100 μg ml^−1^; kanamycin, 100 μg ml^−1^; chloramphenicol, 12.5 μg ml^−1^. For selection of *Streptomyces* transformants, the final antibiotic concentrations were, kanamycin, 50 μg ml^−1^ in MS for *S.* *coelicolor* and 20 μg ml^−1^ in AS-1 for *S. roseosporus*; apramycin, 50 μg ml^−1^ in MS for *S.* *coelicolor* and 10 μg ml^−1^ in AS-1 for *S. roseosporus*; hygromycin, 50 μg ml^−1^ in MS or AS-1 for *Streptomyces*; nalidixic acid, 25 μg ml^−1^ in MS or AS-1 for *Streptomyces*.

### Construction of plasmids

Of the 15 mutated *attP*-*attB* pairs^17^, *attP_6_*-*attB_6_* was randomly chosen for this experiment. The sequences of *attB_6_* and *attP_6_* were obtained by overlapping PCR. For construction of pSV::*attB_6_*-*act*, a 2.0 kb fragment flanking 5′ end of the *act* gene cluster was amplified from genomic DNA of *S. coelicolor* M145 with primer pair act-Up F/act-Up R. The amplicon was diluted 1:100 and used as templates for the second round of PCR with primer pair attB_6_-in F/act-Up R. The product from the second amplification reaction was diluted again and underwent a third run with primer pair attB_6_-out F/act-Up R. The final product was digested with HindIII/BamHI and then inserted into the corresponding sites of pUC119::*neo* to generate pUC119::*neo*-*attB_6_*-*act*. The origin of transfer (*oriT*) from plasmid RK2 was amplified from pKC1139 with primer pair oriT F and oriT R, subsequently digested with EcoRI and inserted into the EcoRI site of pUC119::*neo*-*attB_6_*-*act* to generate pSV::*attB_6_*-*act* (Fig. S1a). For construction of pKC1139::*attP_6_*-*act*, a 2.0 kb fragment flanking 3′ end of the *act* gene cluster was amplified from genomic DNA of *S. coelicolor* M145 with primer pair act-Dn F/act-Dn R. The amplicon with 1:100 dilution served as templates for the second round of PCR with primer pair attP_6_ F/act-Dn R. The final product was digested with HindIII/EcoRI and then inserted into the corresponding sites of pKC1139 to generate pKC1139::*attP_6_*-*act* (Fig. S1b).

For construction of pSV::*attB_6_*-*nap* and pSV::*attB_6_*-*dap*, a 2.0 kb fragment flanking 5′ end of the *nap* and *dap* gene cluster was amplified from genomic DNA of S. *roseosporus* NRRL 15998 with primer pairs nap-Up F/nap-Up R and dap-UpF/dap-Up R, respectively. The product was digested with XbaI/BamHI and used to replace the 2.0 kb fragment upstream of the *act* gene cluster in pSV::*attB_6_*-*act*. For construction of pKC1139::*attP_6_*-*nap* and pKC1139::*attP_6_*-*dap*, a 2.0 kb fragment flanking 3′ end of the *nap* and *dap* gene cluster was amplified from genomic DNA of S. *roseosporus* NRRL 15998 using primer pairs nap-Dn F/nap-Dn R and dap-Dn F/dap-Dn R, respectively. The product was digested with BamHI/EcoRI and used to replace the 2.0 kb fragment downstream of the *act* gene cluster in pKC1139::*attP_6_*-*act*. To ensure the authenticity of DNA sequences, all PCR products were verified by sequencing.

### Construction of double-cointegrate strains

To insert *attB_6_* and *attP_6_* at sites flanking the *act* gene cluster of *S. coelicolor*, pSV::*attB_6_*-*act* and pKC1139::*attP_6_*-*act* were conjugated into *S. coelicolor* M145. The pSV::*attB_6_*-*act* is unable to replicate alone in *Streptomyces* and selection with kanamycin allows to select exconjugants in which pSV::attB6-act is inserted into the *S. coelicolor* genome. The pKC1139::*attP_6_*-*act* is a derivative of the *E. coli*–*Streptomyces* shuttle vector pKC1139 that contains a *Streptomyces* temperature-sensitive origin of replication from pSG5^15^. When the incubation temperature is higher than 34°C, pKC1139::*attP_6_*-*act* turns into non-replicating plasmid and *attP_6_* was then inserted into *S. coelicolor* genome with selection of apramycin to obtain double-cointegrate strain Sco-actB_6_P_6_. Similar strategy was used for the construction of double-cointegrate strains Sro-napB_6_P_6_ and Sro-dapB_6_P_6_.

### Excision of targeted regions

The integrative plasmid pIJ10500^36^ is a derivative of pMS82 which contains the phage ϕBT1 integrase gene and integrates intragenically into *SCO4848* encoding a putative integral membrane protein^16^. It was conjugated into a randomly selected strain Sco-actB_6_P_6_, Sro-napB_6_P_6_ and Sro-dapB_6_P_6_, respectively. The exconjugants were initially selected with hygromycin and ten randomly chosen exconjugants were passed twice on MS or AS-1 plates supplemented with kanamycin and apramycin, and subject to genomic and plasmid extraction. Excision of targeted region from *Streptomyce* genome was analyzed by PCR amplifications using genomic DNA templates and primer pairs B_6_-VF/actDn-VR, B_6_-VF/napDn-VR and B_6_-VF/dapDn-VR. In the meantime, PCR amplifications were performed with plasmid DNA template by using primer pairs P_6_-VF/actUp-VR, B_6_-VF/napUp-VR and B_6_-VF/dapUp-VR. Strains with excision of the targeted regions were designated as M145-MCact, Sro-MCnap and Sro-MCdap, respectively.

### Deletion of the antibiotic gene clusters from *Streptomyces*

A single colony of M145-MCact was randomly chosen and passed three times on nonselective MS plates at 28 °C. Spores were harvested, serially diluted, and then spread on MS agar. After growing for 4 days at 40°C, colonies were replicated on MS agar plates containing kanamycin or apramycin. Strains lacking the *act* gene cluster (M145-Dact) are apramycin sensitive (Apr^s^) and kanamycin resistant (Kan^r^). Apr^s^ and Kan^r^ strains were further verified by PCR. In a similar way, strains lacking the *nap* and *dap* gene clusters (Sro-Dnap and Sro-Ddap) were obtained by the removal of pKC1139::*nap* and pKC1139::*dap* from Sro-MCnap and Sro-MCdap.

### Actinorhodin quantification

To quantitate actinorhodin production, *S. coelicolor* M145 and its derivatives were grown in 50 ml of R5MS at 28°C. 1 ml culture was harvested in a time-course and treated with KOH (1 N final concentration), and titer was calculated by measuring the absorbance at 640 nm^37^.

### Production and analysis of daptomycin

Small-scale fermentation of daptomycin was carried out by following the procedures described previously^38^,^39^ with minor modifications. In brief, starter culture was grown in TSB for 48 h, 1 ml of starter culture was transferred to A355 (1% [wt/vol] glucose, 1.5% [vol/vol] glycerol, 1.5% [wt/vol] soya peptone, 0.3% [wt/vol] NaCl, 0.5% [wt/vol] malt extract, 0.5% [wt/vol] yeast extract, 0.1% [vol/vol] Tween 80 and 2% [wt/vol] MOPS, pH 7.0) and grown for 36 h as seed culture, and 1 ml of seed culture was transferred into a shake flask containing 50 ml A346 (1% [wt/vol] glucose, 2% [wt/vol] soluble starch, 0.5% [wt/vol] yeast extract, 0.5% [wt/vol] casein and 4.6% [wt/vol] MOPS, pH 7.0). The cultures were incubated for different time points at 28°C before fermentation broths were collected by centrifugation.

For daptomycin analysis, culture broths were centrifuged at 13,000 × g for 10 min to remove the mycelia. The supernatants were filtered through a Millipore membrane (pore diameter, 0.22 μm) and 50 μl of sample was used for HPLC analysis. Separation of daptomycin was achieved with an Agilent 1100 HPLC system and a ZORBAX SB-Aq column (5 μm pore size, 4.6 by 250 mm). HPLC conditions were described as follows: gradient elution with buffer A (0.01% [vol/vol] trifluoroacetic acid in acetonitrile) and buffer B (0.01% [vol/vol] trifluoroacetic acid in ddH_2_O), flow rate at 1.0 ml/min, ultraviolet detection at wavelength of 224 nm. The elution profile was a linear gradient of 10%–100% buffer A over 22 min, a hold at 100% buffer A over 3 min, a linear gradient of 100%–10% buffer A over 2 min and a final hold at 10% buffer A over 3 min.

Bioassay against *S. aureus* was performed as previously described with modifications^38^. In brief, *S. roseosporus* and its derivatives were patched on AS-1 agar. After incubation for 2–5 days at 28°C, agar plugs were prepared from the patches, placed on the surface of an empty Petri dish, and overlaid with culture of indicator strain in soft nutrient agar containing 5 mM CaCl_2_. The zone of inhibition was assessed after overnight incubation at 37°C.

## Author Contributions

D.D. and W.L. performed the experiments. T.Y. assisted with design of the project. L.H. assisted with the primary data analysis. N.G. conceived and designed the project, and wrote the manuscript. T.H. supervised the project and revised the manuscript.

## Supplementary Material

Supplementary Information Supplemental Information

## Figures and Tables

**Table 1:** Strains and plasmids used in this study

Strains/plasmids	Genotype/description	Reference/source
***E. coli***		
Top10	F^–^ *mcrA* Δ(*mrr*-*hsdRMS*-*mcrBC*) Φ80*lac*ZΔM15 Δ*lacX74* *recA1* *araD139* Δ(*ara leu*)*7697* *galU* *galK* *rpsL* (StrR) *endA1 nupG*	Invitrogen
ET12567	F^−^ *dam-13*::Tn*9 dcm-6 hsdM hsdR zjj-202*::Tn*10 recF143 galK2 galT22 ara-14 lacY1 xyl-5 leuB6 thi-1 tonA31 rpsL136 hisG4 tsx-78 mtl-1 glnV44*	40
***Staphylococcus***		
*S. aureus*	A indicator strain	41
***Streptomyces***		
*S. coelicolor* M145	Prototrophic; SCP1^−^ SCP2^−^ Pgl^+^	32
*S. coelicolor* M1146	Δact Δred Δcpk Δcda	31
Sco-actB_6_P_6_	A derivative of *S. coelicolor* M145 with *attB_6_* and *attP_6_* flanking *act* gene cluster	This study
*S. roseosporus* NRRL 15998	A daptomycin-producing strain	Broad Institute
Sro-pKC1139	A derivative of *S. roseosporus* NRRL 15998 containing pKC1139	This study
Sro-napB_6_P_6_	A derivative of *S. roseosporus* NRRL 15998 with *attB_6_* and *attP_6_* flanking *nap* gene cluster	This study
Sro-dapB_6_P_6_	A derivative of *S. roseosporus* NRRL 15998 with *attB_6_* and *attP_6_* flanking *dap* gene cluster	This study
M145-Dact	Δact	This study
M145-MCact	A derivative of *S. coelicolor* M145 containing multicopy of *act* gene cluster	This study
M1146-pKC1139	*S. coelicolor* M1146 containing pKC1139	This study
M1146-MCact	*S. coelicolor* M1146 containing pKC1139::*act*	This study
Sro-MCnap	A derivative of *S. roseosporus* NRRL 15998 containing multicopy of *nap* gene cluster	This study
Sro-MCdap	A derivative of *S. roseosporus* NRRL 15998 containing multicopy of *nap* gene cluster	This study
**Plasmids**		
pUZ8002	*tra neo* RP4	42
pUC119::*neo*	pUC119 containing kanamycin resistance gene (*neo*)	43
pKC1139	*E.coli*-*Streptomyces* shuttle plasmid contains a *Streptomyces* temperature-sensitive origin of replication	15
pIJ10500	A derivative of pMS82 containing ϕBT1 integrase gene	36
pUC119::*neo*-*attB_6_*-*act*	A derivation of pUC119::*neo* containing *attB_6_* and 2.0 kb homologous region flanking the 5′ end of *act* gene cluster	This study
pSV::*attB_6_*-*act*	A derivation of pUC119::*neo* containing the origin of transfer (*oriT*) from plasmid RK2, *attB_6_* and 2.0 kb homologous region flanking the 5′ end of *act* gene cluster	This study
pSV::*attB_6_*-*nap*	A derivation of pUC119::*neo* containing the origin of transfer (*oriT*) from plasmid RK2, *attB_6_* and 2.0 kb homologous region flanking the 5′ end of *nap* gene cluster	This study
pSV::*attB_6_*-*dap*	A derivation of pUC119::*neo* containing the origin of transfer (*oriT*) from plasmid RK2, *attB_6_* and 2.0 kb homologous region flanking the 5′ end of *dap* gene cluster	This study
pKC1139::*attP_6_*-*act*	A derivation of pKC1139 containing *attP_6_* and 2.0 kb homologous region flanking the 3′ end of *act* gene cluster	This study
pKC1139::*attP6*-*nap*	A derivation of pKC1139 containing *attP_6_* and 2.0 kb homologous region flanking the 3′ end of *nap* gene cluster	This study
pKC1139::*attP6*-*dap*	A derivation of pKC1139 containing *attP_6_* and 2.0 kb homologous region flanking the 3′ end of *dap* gene cluster	This study
pKC1139::*act*	A derivation of pKC1139 containing 25 kb fragment including actinorhodin gene cluster and its flanking sequences	This study
pKC1139::*nap*	A derivation of pKC1139 containing 45 kb fragment including napsamycin gene cluster and its flanking sequences	This study
pKC1139::*dap*	A derivation of pKC1139 containing 157 kb fragment including daptomycin gene cluster and its flanking sequences	This study
