# Supplementary material for: Genome engineering and direct cloning of antibiotic gene clusters via phage ϕBT1 integrase-mediated site-specific recombination in Streptomyces
Source: Sci Rep. 2015 Mar 4;5:8740. doi: 10.1038/srep08740 (PMC4349145; doi:10.1038/srep08740)
Supplement: Supplementary Information — Supplemental Information [file srep08740-s1.pdf]

Genome engineering and direct cloning of antibiotic gene clusters via phage ϕBT1 integrase-mediated site-specific recombination in *Streptomyces*

Deyao Du[a,b#], Lu Wang[a,c#], Yuqing Tian[a], Hao Liu[c], Huarong Tan[a*], Guoqing Niu[a*]

[a] State Key Laboratory of Microbial Resources, Institute of Microbiology, Chinese Academy of Sciences, Beijing, China

[b] University of Chinese Academy of Sciences, Beijing, China

[c] Key Laboratory of Industrial Fermentation Microbiology, Ministry of Education, College of Biotechnology, Tianjin University of Science and Technology, Tianjin, China

[#] D. D. and W.L. contributed equally to this work.

*Address correspondence to Guoqing Niu, niugq@im.ac.cn, or Huarong Tan, tanhr@im.ac.cn

State Key Laboratory of Microbial Resources, Institute of Microbiology, Chinese Academy of Sciences, Beijing 100101, China

Supplementary Information

Table S1 Primers used in this study

Primer	Sequence (5′ → 3′)	Purpose
oriT F	AATTGAATTCTGGCCAGCTAGCTAGAGTCG	*oriT* cloning
oriT R	AATTGAATTCTGGAATCGCTAGAGCTTGCATG	*oriT* cloning
act-Up F	AATTCTAGATCTTCCCCATGCGGGACA	pSV::attB6-act construction
act-Up R	AATTGGATCCGCCGCGACCTTCACCGAG	pSV::attB6-act construction
act-Dn F	TCTCTGGACACTGATCCATGGGAAACTACTCAGCAGGATCCTCAGCGGCCCGTGGCACC	pKC1139::attP6-act construction
act-Dn R	AATTGAATTCGGACGCGACGCCCCCTCAGGAC	pKC1139::attP6-act construction
attB$_6$-in F	TTGACGAAACTGATCCAGATGATCCAGCTCTAGATCTTCCCCATGCGGG	pSV::attB6-act construction
attB$_6$-out F	AATAAGCTTCCAGGTTTTTGACGAAACTGATCCAGATG	pSV::attB6-act construction
attP$_6$ F	AATAAGCTTTGCTGGGTTGTTGTCTCTGGACACTGATCCATGGG	pKC1139::attP6-act construction
nap-Up F	AATTCTAGATCAGGCGCGGCGGACGATGA	pSV::attB6-nap construction

nap-Up R	AATTGGATCCGGACTTGTCCCCTAGTCAAGCC	pSV::attB6-nap construction
nap-Dn F	AATTGGATCCAAGCCGAGGAGGACCCGCATG	pKC1139::attP6-nap construction
nap-Dn R	AATTGAATTCGTGCGCCTCGTCCTCGACCA	pKC1139::attP6-nap construction
dap-Up F	AATTCTAGAAGACCGTCCGCGCCGGAGGT	pSV::attB6-dap construction
dap-Up R	AATTAGATCTCGTCGGCCCGGAGTACGGCG	pSV::attB6-dap construction
dap-Dn F	AATTGGATCCGAACGGGCCTTGCGGGTGTCG	pKC1139::attP6-dap construction
dap-Dn R	AATTGAATTCCTGGACGTGTCCGGGGCA	pKC1139::attP6-dap construction
B_6-VF	CACACAGGAAACAGCTATGACC	excision confirmation
P_6-VF	GATCGGCACTTTGCATCGGCCG	excision confirmation
actUp-VR	GCGTACTGGAGTGGATGG	excision confirmation
actDn-VR	GCACTACCTCCACACGCC	excision confirmation
napUp-VR	TCGTGTTGTGCGGTGATGTTC	excision confirmation
napDn-VR	GTTCAGCAGTACATCGACAACG	excision confirmation
dapUp- VR	CGCCCATCTGCTGAACTT	excision confirmation
dapDn-V R	AGCGAACTGCTCTCCACCCA	excision confirmation

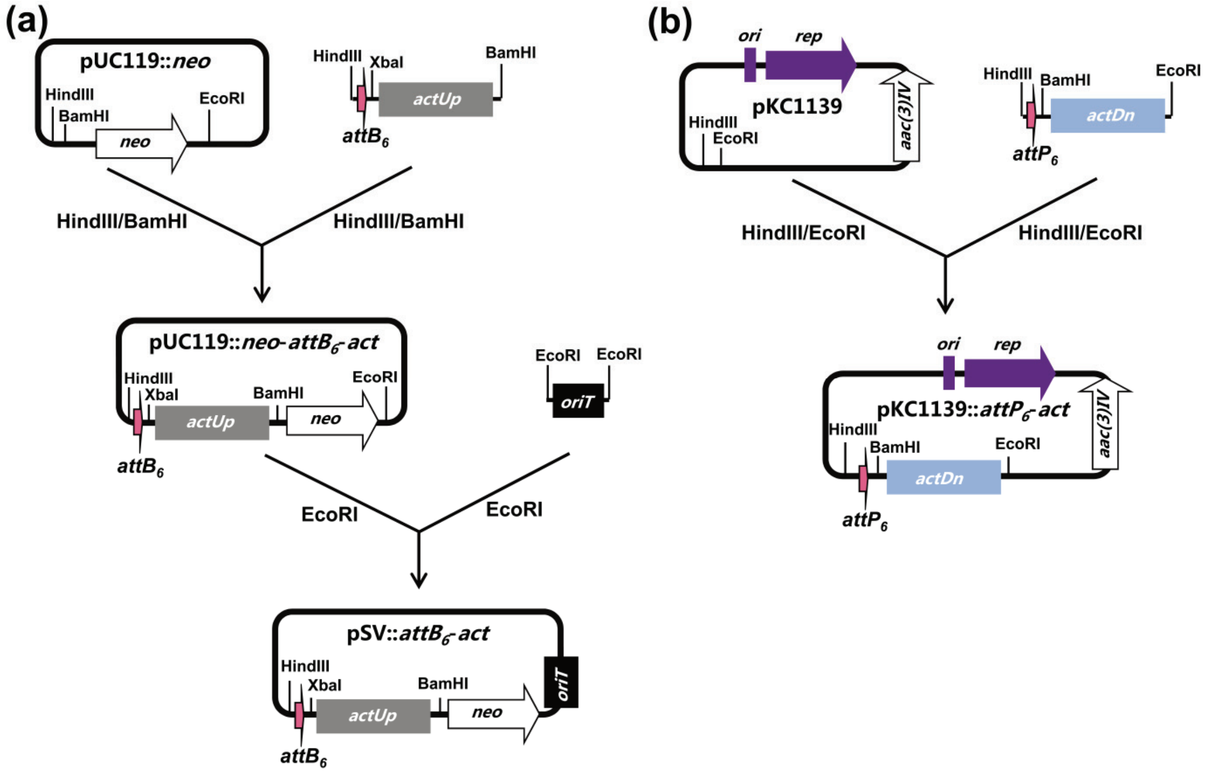

Figure S1. Schematic diagram for the construction of pSV::*attB$_6$-act* (a) and pKC1139::*attP$_6$-act* (b). *neo*: kanamycin resistance gene; *oriT*: the origin of transfer from plasmid RK2; *actUp*: a 2.0 kb fragment flanking the 5′ end of *act* gene cluster; *aac(3)IV*: apramycin resistance gene; *ori*: temperature-sensitive origin of replication from pSG5; *rep*: *rep* encoding a replication initiator protein from pSG5; *actDn*: a 2.0 kb fragment flanking the 3′ end of *act* gene cluster.

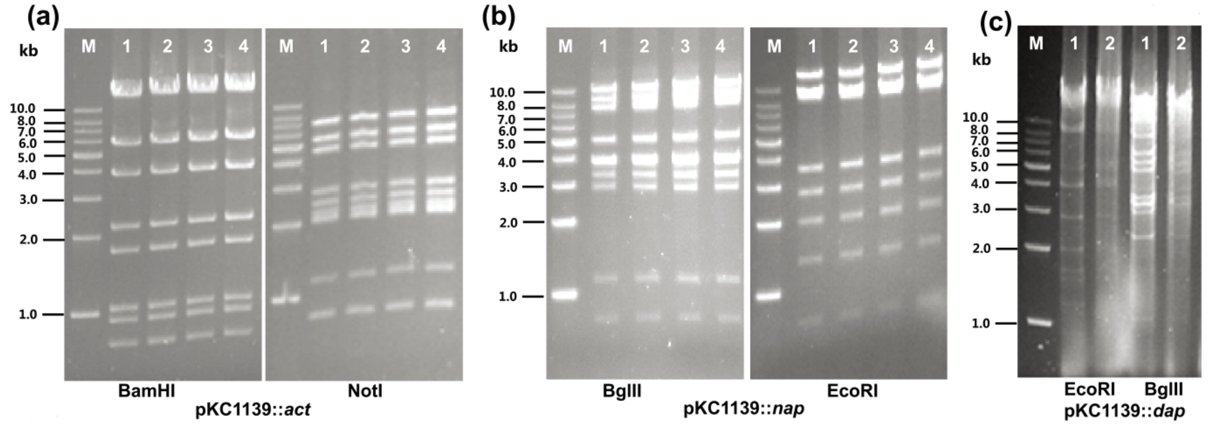

Figure S2. Confirmation of pKC1139::*act*, pKC1139::*nap* and pKC1139::*dap* by restriction digestion. (a) Agarose gel electrophoresis showing pKC1139::*act* digested with BamHI and NotI, respectively. Expected fragment sizes of BamHI digestion are 15222, 5804, 3923, 2172, 1731, 1029, 928 and 743 bp. Expected fragment sizes of NotI digestion are 7136, 5391, 4700, 2767, 2437, 2209,2087, 2018, 1166, 827 and 814 bp. (b) Agarose gel electrophoresis showing pKC1139::*nap* digested with BglII and EcoRI, respectively. Expected fragment sizes of BglII digestion are 10307, 8539, 7714, 5145, 3956, 3910, 3829, 3292, 2849, 1138, and 776 bp. Expected fragment sizes of EcoRI digestion are 14082, 9347, 8860, 8481, 3620, 2724, 1991, 1345, 735 and 270 bp. (c) Agarose gel electrophoresis showing pKC1139::*dap* digested with EcoRI and BglII, respectively. Expected fragment sizes of EcoRI digestion are 57543, 35396, 31107, 16871, 7908, 3883, 2685, 1920, 1563, 1433, 1154, 897, 510 and 455 bp. Expected fragment sizes of BglII digestion are 33236, 22195, 13746, 10869, 9456, 9448, 9385, 9198, 8231, 7470, 5915, 5292, 4856, 3479, 3270, 3183, 2247, 1046, 648, 102 and 53 bp. M: 1 kb DNA Ladder.

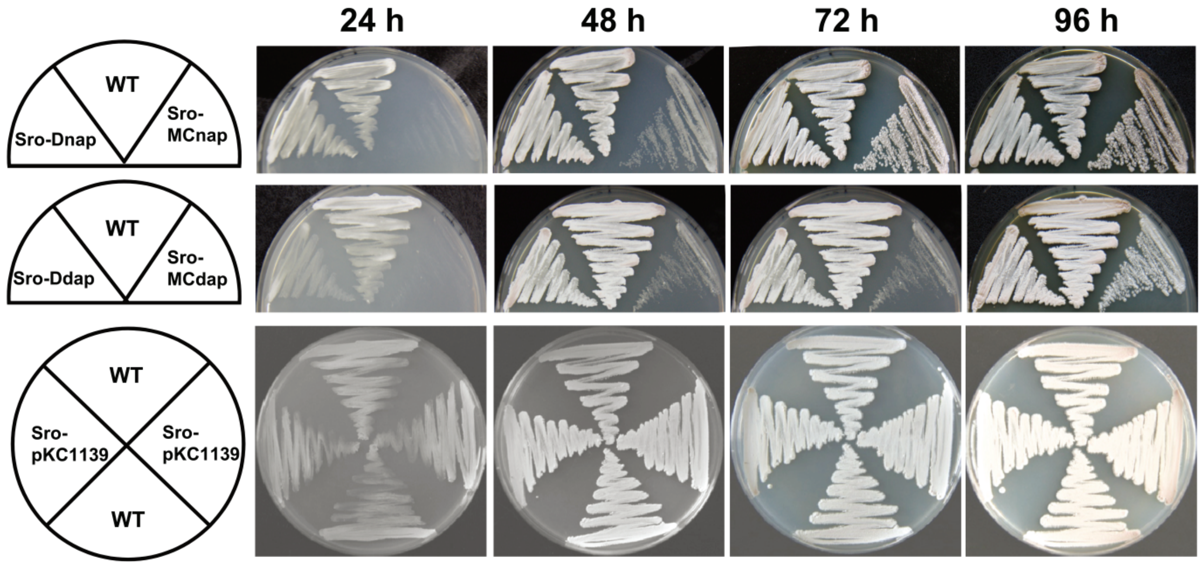

Figure S3. Effect of extra copy numbers of antibiotic gene clusters on growth of *Streptomyces*. Phenotype of *S. roseosporus* NRRL 15998 (WT), Sro-MCnap, Sro-Dnap, Sro-MCdap, Sro-Ddap and Sro-pKC1139 after growth on AS-1 agar media for 24, 48, 72 and 96 h. Representative images of three independent experiments with similar results are shown.

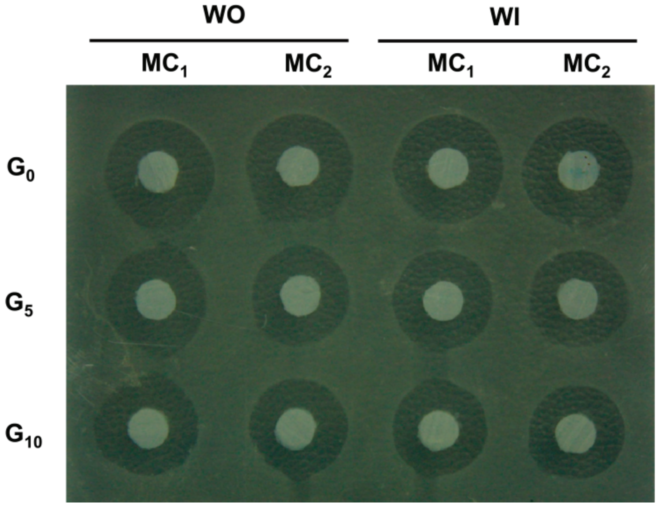

Figure S4. Analysis of daptomycin production in different Sro-MCdap strains. Two randomly chosen strains of Sro-MCdap (MC_1 and MC_2) were passed consecutively for five or ten times on AS-1 plates supplemented with (WI) or without (WO) apramycin. The bioassay against *S. aureus* was performed as in Figure 5. G_0; the original Sro-MCdap strains; G_5, Sro-MCdap passed consecutively on AS-1 plates for five times; G_{10}, Sro-MCdap passed consecutively on AS-1 plates for ten times.
